# Time intervals and associated factors of emergency treatment: first insight into Pakistani system

**DOI:** 10.1186/1755-7682-7-41

**Published:** 2014-09-24

**Authors:** Muhammad Muslim Noorani, Muhammad Farhan Khaliq, Maria Shoaib, Asfandyar Sheikh, Um-E-Roman Moughal, Wardah Moazzum, Syed Arsalan Ali

**Affiliations:** Civil Hospital, Karachi, Pakistan; Dow Medical College, Dow University of Health Sciences, Karachi, Pakistan

**Keywords:** Emergency, Medicine, Civil Hospital, Karachi, Pakistan, Prehospital, Hospital, Delay, Decision time, Transit time, Physician time, Diagnostic time, Transfer time, Chest pain, Abdominal, Respiratory, Neurologic, Urogenital, Musculoskeletal, Trauma, Poisoning, Firearm

## Abstract

The objective of this study is to determine the time interval from decision to seek medical help and arrival of the patients to the emergency department (ED). The duration of stay in ED was also calculated. This study also assesses factors responsible delayed presentation to hospital. This prospective study was conducted during day timings (9 am to 3 pm) from May 2012 to August 2012 in ED at Civil Hospital, Karachi. Patients older than 18 years and meeting the inclusion criteria were considered to be eligible for the study. Statistical analysis was done using SPSS version 17. The study sample consisted of 4,226 patients with a response rate of 96.5%. The median decision time was 146 minutes (IQR = 74–339), median transit time was 84 minutes (IQR = 42–188), median physician time was 58 minutes (IQR = 47–103), median diagnostic time was 148 minutes (IQR = 135–192), median transfer time was 155 minutes (IQR = 118–274) and the median ED LOS was 327 minutes (IQR = 209–488). Patient beliefs regarding spontaneous resolution of the symptoms was the most common reason (44.8%) cited for increased time spent in taking decision to seek medical help. Mode of transportation other than ambulance and traffic gridlock were the most common reasons found to be significantly associated with increased transit time (p < 0.05). The time intervals calculated from our study were found to be higher than studies reported from countries. This calls for urgent intervention for formulation of triage systems to improve patient treatment and satisfaction.

## Introduction

The Accident & Emergency (A&E) department in a hospital is one of the most important players in delivery of healthcare services to patients. Worldwide, Emergency Departments (ED) are reportedly serving increasing numbers of patients who present with complains of variable natures. It has been demonstrated that about 50% of the visits to the ED are for non-emergency reasons[1–5]. This large patient census with variable complains dictates the necessity of classification of patients according to the severity of symptoms. This issue has been adequately addressed by development of emergency triage systems in different countries where it has eased the burden on emergency departments[6]. The triage system takes into account patient requirements of healthcare services and allocates valuable finite resources available to the A&E departments to those who require it the most[6, 7].

The triage system has been found to be independently associated with decreased length of stay (LOS) in emergency department[8, 9]. Emergency LOS is an independent predictor of Inpatient LOS[10]. Thus decreased inpatient LOS due to implementation of triage is related to rapid treatment of patients with severe symptoms. The shorter inpatient LOS also prevents access block and indirectly improves the efficiency of emergency departments. Whereas the use of triage systems is commonplace in developed countries, developing countries have yet to develop and integrate such a system within their practice[11]. The current state of emergency medicine in Pakistan leaves a lot to be desired. Lack of basic healthcare facilities, poor funding status of the hospital departments by state and lack of triage system in most centers are important problems in emergency medicine. Hospitals with no triage systems generally follow the “first come-first served” pattern of provision of healthcare services. This system neither takes into account the severity of symptoms of the patients nor fully assesses the relative needs of patients requiring treatment. This often leads to patients with severe underlying conditions waiting for their turns in the populous A&E department.

The purpose of conducting this study is to determine the current pre-hospital timing patterns of the patients and the time spent in A&E department. We also aim to determine factors responsible for delayed presentation to the ED. This study is a pioneer study as it aims to broadly assess the time intervals and factors important in healthcare provision. This study will enable us in critical analysis of the prevalent conditions and the will highlight the need of development of a triage system for our setting.

## Methods

This descriptive prospective study was conducted from May 2012 to August 2012 in Accident & Emergency (A&E) department, Civil Hospital, Karachi (CHK). This teaching hospital is associated with Dow University of Health Sciences and is a 1900 bed tertiary healthcare centre. The A&E department has an annual census of >400,000 patients. This department is well staffed 24 hours a day by certified physicians and trainee doctors. The patients visiting our setting belong to the low socioeconomic strata with most of the patients earning below Pakistani Rupees (PKR) 10,000 (~ <120 USD). These patients come from all parts of Sindh seeking free treatment from experienced doctors. This hospital provides free treatment to all patients seeking medical help. This is responsible for the higher patient volume as compared to other centers.

**Figure 1:**
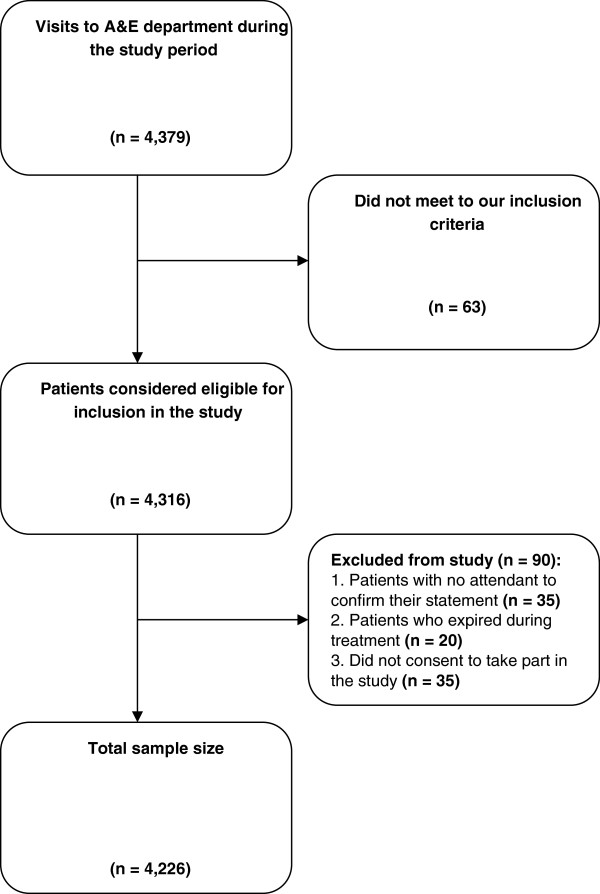
**Illustration of patient time intervals.**

**Figure 2:**
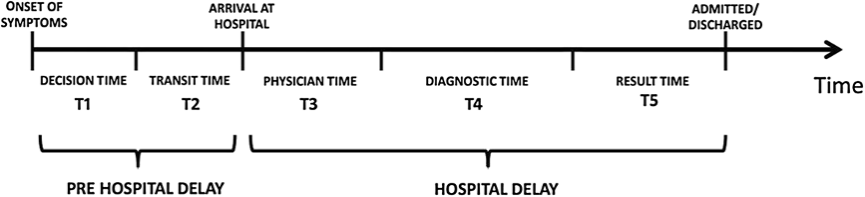
**Patient recruitment diagram.**

All adult patients over the age of 18 years who visited our A&E department were considered to be eligible for participation in our study. *See* Figure 1*for Patient recruitment diagram, and* Figure 2*for a pictorial depiction of different delays*. A separate department that deals with pediatric emergencies exists at our hospital; hence younger patients were not included in our study. The inclusion criteria were decided after conducting a preliminary study to determine the common presenting complains at our centre. We classified these complains into 9 categories. These categories were chest pain, abdominal complains (abdominal pain, acute abdomen, colic, vomiting, hematemesis, diarrhea, jaundice), respiratory complains (asthma, breathlessness, pneumonia, chronic obstructive pulmonary disease, pleural effusion, pneumothorax, aspiration), musculoskeletal complains (joint pain, muscle cramps, non-traumatic fractures), uro-genital disorders (burning micturition, urodynia, urinary discharge, urinary retention, oliguria, hematuria, anuria), neurological complains (fits, delirium, headache, meningitis), all cases of poisoning, firearm injuries and trauma injuries (RTA’s, fractures, lacerations). Patients who refused to take part in the study, patients reporting outside the study time and patients with no attendants to confirm their statement and those who died while being transported and those who expired during treatment in the ED were excluded from the study.

This prospective study employed a questionnaire tool to collect information from patients. The investigators were present in the A&E department from 9 am to 3 pm on all days except Saturdays and Sundays. Consecutive sampling of the incoming patients was done. The questionnaires for this study were filled by investigators in the ward, after seeking informed oral consent from the patients. Patients were assured that their details and responses will remain confidential. In order to ensure that the information provided by the patient is not subject to recall bias, all information provided by the patients was confirmed by their attendants. After the culmination of interview, a verbal reiteration of the responses provided by the patients was done to verify the responses provided. These patients were asked about the time delay in their decision to seek help, and the time each patient had spent in commuting to the hospital. A further confirmation of patient’s decision to seek help was sought from the attendant. A confirmation of the time spent in the vehicle used by patient for commuting to the hospital was done by asking the driver of the vehicle, when possible. The patients who made up the study sample were followed by the investigators periodically to determine their progress in the emergency room.

We find it pertinent to mention here two special cases. For all cases of suspected MI, patients were referred to the cardiac emergency after a rapid preliminary assessment by the main A&E department. The cardiac emergency is located within the Department of Cardiology in our hospital. This ensures provision of timely life-saving measures to such high-risk patients. In the cardiac emergency, our investigators followed the incoming patients during the process of their arrival, their physician consult and the diagnostic testing of the patients. This diagnostic testing includes Point of Care Tests (POCT) for cardiac biomarkers and other serum markers which were sent to the central lab. Periodic follow-up of A&E visitors was done to ensure the accuracy in assessment of time period at various stages of treatment. Patients presenting with renal or urinary symptoms were referred to the department of Sindh Institute of Urology and Transplantation (SIUT) after a provisional diagnosis was formed for the problem. These patients were also followed during the process of confirmatory diagnosis and the transfer of the patients to the SIUT ward.

The study tool was designed after extensive literature review on the subject. The provisional questionnaire consisted of open ended questions which were aimed to assess the general pattern of presenting complains and the timings related to treatment. A final instrument was provisioned for use after review of the responses received from the patients. Suggestions from the faculty members of departments of Accident and Emergency medicine and Community Medicine were considered in this process. This final questionnaire was pre-tested on 40 random patients presenting with different complains in the A&E department. The value of Cronbach’s alpha for the final questionnaires was greater than 0.7.

The questionnaire consisted of 4 sections. The first section consisted of demographic information of the patient. It included information about the age, gender, ethnicity, educational level and socioeconomic status, area of residence, patient residence type (personal home, shared living or homeless). It also included information about co-morbid conditions of the patient, history of physician visits and past visits made to the A&E departments. The second section dealt with the presenting complains of the patients and other associated information. It included questions regarding the reasons for visit to the A&E department, past history of similar symptoms, perception of symptoms and measures undertaken to relieve them. It also included questions about arrival of patients through referral, mode of transportation and general state of consciousness at the time of presentation. The third section of the questionnaire consisted of questions regarding the pre-hospital intervals of the patients. Questions about the passage of time since onset of symptoms to seek help and the time spent in transit were a part of this section. It also dealt with reasons associated with pre-hospital delay. The fourth section dealt with the hospital delay faced by the patients. It had questions which assessed the time duration since patient arrival to the hospital A&E wing and the first checkup by physician, and the time duration between patient arrival and the first investigation ordered for patient. The time interval between result of investigations and the transport of patient to relevant facility (ward or ICU) or patient discharge was also assessed in this section.

The study protocol was reviewed and accepted by the Institutional Review Board (IRB) at the Dow University of Health Sciences. A permission to conduct study was granted by the Medical Superintendent (MS) of the Civil Hospital, Karachi. Sub-heading Operational definition gives the operational definitions for this paper. Statistical Package for Social Sciences Windows version 17 was used for database assembly and analysis. Only those questionnaires were included which were completed. Descriptive analysis (means, standard deviations and percentages) was performed. To determine significant associations between variables, cross-tabbing of the variables was performed and Pearson Chi squared test was applied. Values were considered significant when they were below 0.05 (p < 0.05).

**Operational definitions**

In order to assess the duration of time intervals in the course of treatment of patients, our study utilized two major divisions. These were:

1. **Pre Hospital Delay:** This time interval represents the time period from the development of symptoms and the arrival of the patient to the A&E department. This is further divided into:
  - **Decision time:** This represents the time interval between the onset of symptoms in a patient and the decision of the patient to seek help
  - **Transit time:** This represents the interval of time which the patient spent in commuting from the place where his symptoms developed to the healthcare facility.
2. **Hospital Delay:** This time interval represents the time from patient registration at the A&E counter till the end of his treatment process (this includes discharge from the hospital, hospital admission and patient departure against medical advice.)
  - **Physician time:** This time interval represents the time period between arrival of patient into the A&E department and the first consult made by a doctor.
  - **Diagnostic time:** This represents the time interval between the physician order of diagnostic tests and the arrival of the results of the same.
  - **Transfer time:** This time interval was noted from the hospital records and represents the interval from the diagnosis of the condition to any of these four alternate endings:
    1. Treatment and Discharge of the patient
    2. Patient admitted into the hospital
    3. Referral to another hospital
    4. Patient’s departure against medical advice

**Length of Stay:** The length of stay (LOS) refers to the time sum of all three conditions stipulated by the hospital delay.

## Results

We included 4,226 patients in the study with a response rate of 87.02%. The mean age of patients was 40.9 ± 17.9 years (range = 18–92 years). Most (51.3%) of the cases were male while 48.69% were female with a male to female ratio of 1.05:1. A sizable majority (46.2%) of patients were illiterate. Amongst all ethnic groups visiting the A&E department, Muhajirs represented the group utilizing the A&E services at our hospital the most (31.5%). Most of the study participants hailed from low Socioeconomic status (SES) background with 1445 (34.2%) of the respondents earning less than PKR 5,000 per month (~USD 50/month). Most (88.2%) patients in the study sample were residents of Karachi. Most (91.6%) cases reported that they had their own homes whereas 43 cases (1.01%) reported that they were homeless. A regular visit to the physician was reported by only 1,230 (29.1%) patients. The most common co-morbid conditions in our study sample was hypertension with 681(16.1%) patients followed by diabetes 556(13.15%) and smoking 245(5.79%). A visit to the emergency department at least once in the past 6 months was confirmed by 786 (18.6%) of the cases with 32.9% patients in the same group affirming visiting emergency services more than once in the same time duration. *A summary of patient demographics is presented in* Table 1*.*

**Table 1:** **Demographics of the participants**

Demographics		n	%
Gender	Male	2168	51.3
	Female	2058	48.7
Education	Illiterate	1951	46.2
	Less than 5th grade	646	15.3
	Less than 10th grade	839	19.9
	More than 10th grade	790	18.7
Income	>5000	1445	34.2
	5001-10,000	1006	23.8
	10,001-15,000	1200	28.4
	<15,000	575	13.6
Ethnicity	Sindhi	1072	25.4
	Balochi	566	13.4
	Pathan	580	13.7
	Punjabi	383	9.1
	Muhajir	1333	31.5
	Other	292	6.9
City	Karachi	3726	88.2
	Hyderabad	108	2.6
	Sukkur	28	0.7
	Areas of Sindh other than Karachi, Hyderabad & Sukkur	242	5.7
	Other provinces	122	2.9
Place Living in	Personal Home	3871	91.6
	Sharing Residence	307	7.3
	Hostel	24	0.6
	Homeless	24	0.6
Living with	Family	3988	94.4
	Friends	203	4.8
	Alone	35	0.8

**Figure 3:**
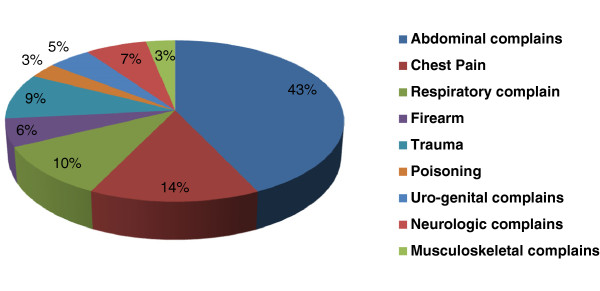
**Percentage of complains included in the study.**

Abdominal pain was the most common (42.9%) presenting complaint in our study sample followed by chest pain (14.33%), respiratory complaints (10.53%) and trauma cases (9.18%). Figure 3*shows the relative frequencies of each presenting complain*. A past history of symptoms that were similar to the presenting symptoms was reported by 1935 (45.7%) patients. In most cases, the symptoms of the disease were recognized by the patients (87.3%). In other cases, family members (8.4%), strangers (2.1%), friends (1.4%) and colleagues (0.7%) recognized the symptoms of disease. Of all the patients presenting to the A&E department, 1247 (29.5%) cases were referred from other hospitals. Patients presenting to the emergency department in unconscious state represented 15.3% of the total sample population. Self-medication to improve the symptoms was reported by 1652 (39.09%). The most common mode of transport for transport to the A&E department was Rickshaws (29.2%). Other modes of transport utilized were Taxi (23.8%), private car (11.19%), and public bus (5.4%). Only 879 patients (20.8%) reported using ambulance services for coming to the hospital.

The factors responsible for increased pre-hospital timings were categorically divided into two groups, those affecting decision to seek medical help and those affecting the patient transit time. See Table 2 for distribution of factors. The median time elapsed since the onset of symptoms to decision to seek medical help was 146 minutes (IQR = 74–339). This was found to be significantly associated with female gender, education level, primary complaint (p < 0.05). Diabetes, hypertension, Chronic Obstructive Pulmonary Disease (COPD) and smoking were found to be significantly associated with the patient reporting time to the A&E department (p < 0.05). The assumption that the symptoms produced by the underlying disease would go away by themselves was reported as the cause of delayed decision to seek medical assistance by 1893 (44.8%) of the patients (p < 0.05). Other patients reported resorting to self-medication (39.09%), did not realize the severity of symptoms (11.2%) and the high cost of transport (15.09%) as important factors in affecting the decision to seek help for their condition (p < 0.05). Other patients reported fear of treatment (21.5%), concern relieved by a family member or colleague (5.06%) and living alone (4.47%) as causes responsible in their decision to get a medical consult. The median time spent in commuting to the hospital was found to be 84 minutes (IQR = 42–188). We found that the choice of mode of transport was found to be significantly associated with the delayed presentation to the emergency department (p < 0.05). Other responsible factors highlighted by the patients include traffic on the roads (37.2%), availability of transportation (14.36%) and long commuting distance from the place where patient decided to seek help to the A&E department (11.07%). These factors were significantly associated with the increased transit time reported by the patients (p < 0.05). Other factors indicated by patients were poor law and order situation prevailing in the city (5.82%) and the poor condition of roads (4.87%). The assumption that the symptoms produced by the underlying disease would go away by themselves was reported as the cause of delayed decision to seek medical assistance by 1893 (44.8%) of the patients (p < 0.05). Other patients reported resorting to self-medication (39.09%), did not realize the severity of symptoms (11.2%) and the high cost of transport (15.09%) as important factors in affecting the decision to seek help for their condition (p < 0.05). Other patients reported fear of treatment (21.5%), concern relieved by a family member or colleague (5.06%) and living alone (4.47%) as causes responsible in their decision to get a medical consult.

**Table 2:** **Factors affecting patient reporting time to the A&E department**

Factors		N (%)	P value
**Decision factors**	**Symptom would go away by itself**	1893 (44.8%)	<0.05
	**Self medication to relieve symptoms**	1652 (39.09%)	<0.05
	**Fear of treatment**	909 (21.5%)	-
	**Neglected the consequences of symptoms**	477 (11.2%)	<0.05
	**Concern relieved by individuals close to the patient**	214 (5.06%)	-
	**Expensive transport**	638 (15.09%)	<0.05
	**Living without friends or family**	189 (4.47%)	-
**Transit factors**	**Traffic/gridlock**	1572 (37.2%)	<0.05
	**Long commuting distance**	468 (11.07%)	<0.05
	**Mode of transport (other than ambulance)**	2850 (67.43%)	<0.05
	**Availability of transportation**	607 (14.36%)	<0.05
	**Poor law and order situation in the city**	246 (5.82%)	-
	**Poor infrastructure of roads**	204 (4.87%)	-

p values were considered significant when below p < 0.05.

The median hospital time interval (length of stay) was calculated to be 327 minutes (IQR = 209–488). The median time since the patient registration at the A&E department and the first contact with physician (Physician time) was calculated to be 58 minutes (IQR = 47–103). Physician time was found to be significantly associated with the age, gender, ethnicity and the presenting complain of the patient (p < 0.05). The median diagnostic time was calculated to be 148 minutes (IQR = 135–192). The primary complain and the choice of diagnostic test were two factors found to be significantly associated with diagnostic time (p < 0.05). The median transfer time was calculated to be 155 minutes (IQR = 118–274) and was found to be significantly associated with the age, primary complain and the diagnostic time of the patients (p < 0.05). See Table 3 for complain wise breakdown of pre-hospital and hospital timings in minutes.

**Table 3:** **Complain wise breakdown of pre-hospital and hospital timings in minutes**

Complains	Pre-Hospital timings		Hospital timings			
	Decision time	Transit time	Physician time	Diagnostic time	Transfer time	Length of stay
Chest pain	132	85	15	20	12	58
	(72–298)	(40–198)	(10–25)	(15–25)	(8–30)	(50–95)
Abdominal	155	90	66	170	210	425
	(83–349)	(45–210)	(50–105)	(145–195)	(140–320)	(212–545)
Respiratory	136	73	54	162	185	384
	(76–288)	(45–220)	(52–98)	(150–185)	(125–290)	(234–610)
Musculoskeletal	1182	95	82	185	110	315
	(725–2380)	(45–185)	(64–132)	(135–220)	(72–155)	(190–362)
Urogenital	450	85	64	158	45	262
	(252–648)	(52–203)	(50–112)	(140–186)	(35–60)	(238–310)
Neurological	176	97	50	165	148	390
	(120–395)	(55–187)	(44–92 )	(130–240)	(90–176)	(175–436)
Poisoning	65	75	35	15	275	315
	(45–130)	(45–152)	(28–52)	(10–20)	(151–380)	(220–575)
Firearm	20	55	12		8	15
	(15–58)	(35–85)	(10–22)	-	(5–11)	(10–25)
Trauma	30	62	48	25	105	266
	(25–70)	(40–105)	(25–42)	(15–55)	(82–245)	(95–330)

Figures were rounded off to the nearest whole number.

## Discussion

Emergency crowding is an ever-growing problem associated with increased patient morbidity and mortality. It has even graver implications when translated to a developing country with limited financial resources. Pakistan, where triage system is yet to be implemented, forms a special case in this scenario. None of the studies previously undertaken comprehensively identifies the reasons responsible for delays in emergency departments. Some of them attempt at merely quantitatively assessing these delays, without placing much focus on the reasons responsible for them, while others have been conducted in limited settings with restricted profiles[12, 13]. This study is the first study of its kind which gives an insight to its readers about the current prevailing conditions that are associated with emergency department. A baseline idea about the time taken to treat acute conditions from the onset of their symptoms along with the factors responsible for prolonging the presenting time to the emergency department can be judged from this study.

The demographic characteristics of study sample represent the low SES patients as our hospital provides free of cost emergency and in-patient healthcare services to all patients. Low SES patients have been demonstrated to be at a higher risk of mortality[14–16]. This patient group requires that special attention be paid to its health needs to decrease the subsequent morbidity and mortality from the acute incidents. Our study indicated that site of residence is significantly associated with transit time and it takes much longer for patients to reach emergency department who live outside the catchment area. This increases the risk of mortality by 1% with every 10kilometers increase[17]. In our study population, about 12% of the population visiting our A&E department was from outside Karachi. The state of road infrastructure in Karachi is not up to the par with existing standards. The condition of road infrastructure has been outlined as a nuisance in transport of patients towards the healthcare facilities by Channa R et al.[18]. The low-income patients travelling from areas outside Karachi have no alternate mode of transport other than roads. The same problems of poor road conditions are faced by patients living within the city. The visitors to our centre most frequently used rickshaws and taxis. Ambulances ranked third in the mode of transportation utilized to reach the hospital. This public mindset of favoring transport modalities other than ambulances has been documented in literature[19]. The decreased use of ambulances has been attributed to poor knowledge of the patients and their attendants regarding the severity of symptoms and the inefficiency of the ambulance services.

Prior visit to the emergency department in the past 6 months was reported by almost 20% of our study population. Out of these more than one third of the respondents reported visiting emergency services more than once in past six months. Several factors can be held responsible for the increased frequency of visit to the emergency visit. Demographic factors like low SES, low education level, marital status of the patient (single or divorced) have been incriminated in increased utilization of emergency services[20–22]. The referral of patients from other health service providers also contributes to the high patient census at our centre. Almost one third of our total sample reported being referred to out center from some other source. We believe that this can be due to several causes. Most of the hospital scattered throughout the country are often ill-equipped and under-staffed to make a favorable impact on the health of the patients. This results in referrals to other centers where services are expected to be better. However in most public sector hospitals, there exists room for improvement in terms of equipment and administrative management. The role of Primary Care Physicians (PCP) in increased volume at the emergency services is important to consider at this point. The ratio of 0.47 doctors to 1,000 patients speaks volumes about the need for doctors in our country[23]. Most patients seek help from PCP’s regarding their problems. A shortage of PCP is a valid reason for increased number of visits to the emergency department by the patients[24–26]. Other factors considered responsible for increasing the emergency department census unnecessarily are patient dissatisfaction with their PCP and the utilization of emergency healthcare services for non-emergency services[27–31].

Abdominal complaints formed the bulk of presentations in our sample. Graff et al. estimated abdominal complaints to comprise 8% of the 100 million ED visits each year[32]. It must be noted here that a large proportion of those who present with abdominal complaints, do not require urgent care[24, 33]. This is also reflected in the exaggerated length of stay seen in these patients. Abdominal complaints were followed by complaints of chest pain. These include those resulting from myocardial infarction and angina pectoris. These patients are drastically affected by delays experienced in emergency departments, many of which can prove to be fatal. Similarly, firearm injuries formed a significant proportion of the patients, which can be attributed to the poor law and order situation in Karachi This problem has haunted the residents of Karachi for quite some time, with more than 500 people killed in acts of violence each year. Several areas are severely affected by strikes called by various factions in the city and riots preceding and succeeding these strikes. All these factors combined cause a hindrance for a common man whose life depends on timely visit to the emergency department.

The pre-hospital time interval was divided into decision time and the time spent in transit to hospital. We find that no studies have been conducted which take into account the pre-hospital timings of patients reporting with all systemic complains. A comparison of our pre-hospital time intervals for specific symptoms with results of our peers is warranted. The median pre-hospital time for patients presenting with most common complains was found to be in a comparable range with our peers[34–37]. The findings of our study regarding significant association of the pre-hospital delay with female gender, education level, nature of the primary complain and the mode of transportation used are similar to the results demonstrated by studies conducted elsewhere[38–42]. In our society, women are more often than not limited to household chores and their movement outside their homes is discouraged. This reason coupled with poor educational status of most women makes it difficult for them to decide when to seek proper medical help for the symptoms of diseases ailing them. Another important determinant of the increased pre-hospital time was found out to be the mode of transport utilized by patients. Not only does a visitor to the emergency department has to brave pot-holed roads which severely impact the patient transit time, but he has other concerns too. These include the traffic on the roads of Karachi, which is the largest city of Pakistan and other factors like long commuting distance and the availability of transportation.

## Limitations

This study took place in a single center public sector tertiary care hospital with most patients from lower socioeconomic status and low level of education. Our study revealed that most of the study cases were residents of Karachi only a small number of patients visited from outside of Karachi. This restricts the generalization of study results to areas outside Karachi because the pattern of utilization of emergency services can be very different from or results. Furthermore, due to use of consecutive sampling technique, the generalization of the study findings is restricted. The use of probability sampling and involvement of multiple hospitals (public and private) would have yielded results that would be more indicative of the prevailing situation.

The selection of timings to observe and follow the patients was decided on while keeping in mind the inflow pattern and logistic factors; we believe that a round the clock patient selection and inclusion pattern will be more representative of the time patterns and the factors responsible for pre-hospital delay. Furthermore our study only involves those patients who managed to arrive to the emergency department and managed to get treatment for their symptoms. The exclusion criteria hinders us from assessing the timing patterns and reasons for delay of patients who died while on the way or died while undergoing treatment in the emergency department. Another limitation to our study is the targeted approach of using nine groups of symptoms and classifying patients according to them. There is a fair chance that the individuals not deemed fit for the study might present another perspective of the underlying problems.

## Conclusion

The time intervals calculated from our study are higher than those reported by other studies. The decision to seek medical help was the major variable in determining pre-hospital time interval of the patients. This time interval was found to be more variable than the time a patient spent in travelling to the hospital. The length of stay in the emergency department was found to be significantly higher in our center as compared to the same time intervals at other institutions.

## Recommendations

In light of findings of our study, we would like to propose several measures to improve the prevailing situation of emergency care at our centre and other centers nationwide. Such measures include public education campaigns which are conducted with the aims of promotion of awareness about common medical ailments and their presenting symptoms. These campaigns should also encourage patients to report to the A&E department at the earliest warning symptoms. These campaigns should be specially directed towards those individuals who have been shown to be at higher risk due to delayed presentation at the emergency department. These people include female gender, people with low education level and low SES. Along with such campaigns, the influence exercised by certain people in seeking earlier treatment for their patients by using coercion should be put to stop with provision of proper security by the hospital. We also recommend that measures be taken to ensure that the ambulance service utilized by the patients is cheap and patient friendly. Provision of EKG and other essential services in the ambulance will be an improvement from the most of the current ambulances plying the city roads. Special attention needs to be paid to the road infrastructure to facilitate easier movement of patients to the hospital. All these recommendations are meant to rectify the discrepancies present currently. However to provide a long term solution, we propose that a triage system should be designed keeping in view health problems seen most commonly in the emergency departments in our country. The decision to initiate treatment should be based on severity assessed by the triage system and non urgent cases should be directed to visit ambulatory clinics.

## References

[1] Baker LC, Baker LS (1994). Excess cost of emergency department visits for nonurgent care. Health Aff.

[2] Doobinin KA, Heidt-Davis PE, Gross TK, Isaacman DJ (2003). Nonurgent pediatric emergency department visits: care-seeking behavior and parental knowledge of insurance. Pediatr Emerg Care.

[3] Lee TJ, Baraff LJ, Guzy J, Johnson D, Woo H (2003). Does telephone triage delay significant medical treatment?: advice nurse service vs on-call pediatricians. Arch Pediatr Adolesc Med.

[4] Kalemoglu M, Keskin O, Demirbas S, Ozisik T (2004). Non-urgent patients in an emergency medical service. Rev Med Chil.

[5] Carter AJE, Chochinov AH (2007). A systematic review of the impact of nurse practitioners on cost, quality of care, satisfaction and wait times in the emergency department. CJEM.

[6] FitzGerald G, Jelinek GA, Scott D, Gerdtz MF (2010). Emergency department triage revisited. Emerg Med J.

[7] Robertson-Steel I (2006). Evolution of triage systems. Emerg Med J.

[8] Yoon P, Steiner I, Reinhardt G (2003). Analysis of factors influencing length of stay in the emergency department. CJEM.

[9] Chan TC, Killeen JP, Kelly D, Guss DA (2005). Impact of rapid entry and accelerated care at triage on reducing emergency department patient wait times, lengths of stay, and rate of left without being seen. Ann Emerg Med.

[10] Liew D, Kennedy MP (2003). Emergency department length of stay independently predicts excess inpatient length of stay. Med J Aust.

[11] Qureshi N (2010). Triage systems: a review of the literature with reference to Saudi Arabia. EMHJ.

[12] Rehmani R (2004). Emergency Section and overcrowding in a University hospital of Karachi, Pakistan. Emergency.

[13] Khan A, Zafar H, Naeem SN, Raza SA (2010). Transfer delay and in-hospital mortality of trauma patients in Pakistan. Int J Surg.

[14] Pappas G, Queen S, Hadden W, Fisher G (1993). The increasing disparity in mortality between socioeconomic groups in the United States, 1960 and 1986. N Engl J Med.

[15] Salomaa V, Niemelä M, Miettinen H, Ketonen M, Immonen-Räihä P, Koskinen S, Mähönen M, Lehto S, Vuorenmaa T, Palomäki P, Mustaniemi H, Kaarsalo E, Arstila M, Torppa J, Kuulasmaa K, Puska P, Pyörälä K, Tuomilehto J (2000). Relationship of Socioeconomic Status to the Incidence and Prehospital, 28-Day, and 1-Year Mortality Rates of Acute Coronary Events in the FINMONICA Myocardial Infarction Register Study. Circulation.

[16] Adler NE, Ostrove JM (1999). Socioeconomic status and health: what we know and what we don’t. Ann N Y Acad Sci.

[17] Nicholl J, West J, Goodacre S, Turner J (2007). The relationship between distance to hospital and patient mortality in emergencies: an observational study. Emerg Med J.

[18] Channa R, Jaffrani H, Khan A, Hasan T, Razzak J (2008). Transport time to trauma facilities in Karachi: an exploratory study. Int J Emerg Med.

[19] Razzak JA, Cone DC, Rehmani R (2001). Emergency medical services and cultural determinants of an emergency in Karachi, Pakistan. Prehosp Emerg Care.

[20] Sun BC, Burstin HR, Brennan TA (2003). Predictors and outcomes of frequent emergency department users. Acad Emerg Med.

[21] Andrén KG, Rosenqvist U (1985). Heavy users of an emergency department: Psycho-social and medical characteristics, other health care contacts and the effect of a hospital social worker intervention. Soc Sci Med.

[22] Mandelberg JH, Kuhn RE, Kohn MA (2000). Epidemiologic analysis of an urban, public emergency department’s frequent users. Acad Emerg Med.

[23] Talati JJ, Pappas G (2006). Migration, medical education, and health care: a view from Pakistan. Acad Med.

[24] Petersen LA, Burstin HR, O’Neil AC, Orav EJ, Brennan TA (1998). Nonurgent emergency department visits: the effect of having a regular doctor. Med Care.

[25] Baker Dw SCDBRH (1994). REgular source of ambulatory care and medical care utilization by patients presenting to a public hospital emergency department. JAMA.

[26] Steinbrook R (1996). The role of the emergency department. N Engl J Med.

[27] Sarver JH, Cydulka RK, Baker DW (2002). Usual source of care and nonurgent emergency department use. Acad Emerg Med.

[28] Sempere-Selva T, Peiró S, Sendra-Pina P, Martínez-Espín C, López-Aguilera I (2001). Inappropriate use of an accident and emergency department: magnitude, associated factors, and reasons–an approach with explicit criteria. Ann Emerg Med.

[29] O’Brien GM, Shapiro MJ, Fagan MJ, Woolard RW, O’Sullivan PS, Stein MD (1997). Do internists and emergency physicians agree on the appropriateness of emergency department visits?. J Gen Intern Med.

[30] Gill JM, Reese CL, Diamond JJ (1996). Disagreement among health care professionals about the urgent care needs of emergency department patients. Ann Emerg Med.

[31] Shesser R, Kirsch T, Smith J, Hirsch R (1991). An analysis of emergency department use by patients with minor illness. Ann Emerg Med.

[32] Graff Iv LG, Robinson D (2001). Abdominal pain and emergency department evaluation. Emerg Med Clin North Am.

[33] Afilalo J, Marinovich A, Afilalo M, Colacone A, Leger R, Unger B, Giguere C (2004). Nonurgent emergency department patient characteristics and barriers to primary care. Acad Emerg Med.

[34] Schroeder JS, Lamb IH, Hu M (1978). The prehospital course of patients with chest pain: Analysis of the prodromal, symptomatic, decision-making, transportation and emergency room periods. Am J Med.

[35] Reilly A, Dracup K, Dattolo J (1994). Factors influencing prehospital delay in patients experiencing chest pain. Am J Crit Care.

[36] McGinn AP, Rosamond WD, Goff DC, Taylor HA, Miles JS, Chambless L (2005). Trends in prehospital delay time and use of emergency medical services for acute myocardial infarction: experience in 4 US communities from 1987–2000. Am Heart J.

[37] Schroeder EB, Rosamond WD, Morris DL, Evenson KR, Hinn AR (2000). Determinants of use of emergency medical services in a population with stroke symptoms: the Second Delay in Accessing Stroke Healthcare (DASH II) Study. Stroke.

[38] Meischke H, Eisenberg MS, Larsen MP (1993). Prehospital delay interval for patients who use emergency medical services: The effect of heart-related medical conditions and demographic variables. Ann Emerg Med.

[39] McConnel CE, Wilson RW (1998). The demand for prehospital emergency services in an aging society. Soc Sci Med.

[40] Mock CN, Tiska M, Adu-Ampofo M, Boakye G (2002). Improvements in prehospital trauma care in an African country with no formal emergency medical services. J Trauma Acute Care Surg.

[41] Berglin B, Hartford M, Karlsson T, Herlitz J (1998). Factors associated with pre-hospital and in-hospital delay time in acute myocardial infarction: a 6-year experience. J Intern Med.

[42] Hartford M, Herlitz J, Karlson BW, Risenfors M (1990). Components of delay time in suspected acute myocardial infarction with particular emphasis on patient delay. J Intern Med.

